# Microfracture for full‐thickness chondral lesions of the knee in elite athletes leads to high return‐to‐play rates

**DOI:** 10.1002/ksa.12808

**Published:** 2025-08-13

**Authors:** David J. Haslhofer, Jobe Shatrov, Mary Jones, Wahid Abdul, Arman Motesharei, Simon V. Ball, Andy Williams

**Affiliations:** ^1^ Fortius Clinic FIFA Medical Centre of Excellence London UK; ^2^ Department for Orthopaedics and Traumatology Kepler University Hospital GmbH, Johannes Kepler University Linz Linz Austria; ^3^ Sydney Orthopaedic Research Institute (SORI) at Landmark Orthopaedics St. Leonards New South Wales Australia

**Keywords:** chondral lesions in the knee, elite athletes, microfracture, return‐to‐play (RTP)

## Abstract

**Purpose:**

Injuries to the knee are common in elite athletes and often involve damage to the articular cartilage. Given the high demands of elite sport, full‐thickness articular cartilage defects in the knee can be career‐limiting or threatening. Microfracture can promote cartilage repair, but the resultant mixed fibrocartilaginous tissue is believed to be less resilient than the native hyaline cartilage. Doubts remain as to whether it can withstand prolonged and intensive sporting activity, and it has become less popular. Also, there is a view that microfracture compromises the results of subsequent chondral resurfacing surgery options. The aim of this study was to determine the factors affecting return‐to‐play (RTP) and continued participation in elite sport by athletes after microfracture in the knee.

**Methods:**

A retrospective review of a consecutive series of elite athletes with chondral injuries in the knee treated with microfracture by the lead surgeon between 2011 and 2020 was undertaken. RTP was defined as competing in at least one match at a professional level or national/international level in amateur sport.

**Results:**

Fifty athletes with a mean age of 24.7 years (±4.0 years) were included. Thirty (60%) footballers, 13 (26%) rugby players and 7 (14%) other elite athletes were treated. Forty‐seven (94%) athletes RTP at a mean time of 9.3 months (±4.1 months) with 43 (86%) still playing at 2 years, and of the 44 with 5‐year follow‐up, 24 (54.5%) were still playing. The lateral femoral condyle was the most common location for the chondral lesion (56%). Larger lesions (over 2 cm diameter) significantly reduced RTP (*p* = 0.048), and the ability to continue to play at 5 years (*p* = 0.051 and *p* = 0.002), but this was not significant at 2 years. Multiple lesions significantly reduced playing at 2 and 5 years (*p* < 0.001).

**Conclusion:**

The rate of RTP of professional athletes after microfracture in the knee is high but takes a long time, and the ability to continue playing reduces over time and is affected by the size and number of lesions requiring microfracture.

**Level of Evidence:**

Level IV retrospective cohort study.

**Abbreviations**
ACI: autologous chondrocyte implantation
ACL: anterior cruciate ligament
BMI: body mass index
BMS: bone marrow stimulation
CPM: continuous passive motion
MACI: matrix‐augmented autologous chondrocyte implantation
MLB: Major League Baseball
MRI: magnetic resonance imaging
NBA: National Basketball Association
NFL: National Football League
NHL: National Hockey League
OAT: osteochondral autograft transplantation
PROMs: patient‐reported outcome measures' scores
RTP: return‐to‐play
SD: standard deviation

## INTRODUCTION

Knee injuries are common in elite athletes, and the prevalence of damage to the articular cartilage is up to 36% [9], which is twice as high as for non‐professional athletes under 40 years of age [6]. Most chondral lesions do not need surgery, but those that remain symptomatic can be career‐limiting if not career‐ending [27, 28] in athletes due to the high demands of elite sport and may necessitate surgery.

Options for the surgical treatment of focal cartilage lesions include simple debridement and stabilisation, bone marrow stimulation (BMS) such as microfracture (with or without a scaffold), (matrix‐augmented) autologous chondrocyte implantation (MACI/ACI), scaffolds, osteochondral autograft transplantation (OAT) or osteochondral allograft transplantation [4, 14, 15].

Microfracture involves perforating the subchondral bone, allowing bone marrow to be released with blood, aiming to fill chondral lesions and allow healing [13, 24, 25, 33, 35]. This technique leads to the formation of fibrocartilaginous tissue [12]. However, histological studies have suggested that the new tissue formed by such ‘BMS’ is less resilient and therefore inferior to native hyaline cartilage [7, 18, 29].

Doubts remain as to whether fibrocartilaginous tissue can withstand prolonged and intensive activity, such as sports participation. Previous studies have demonstrated that return‐to‐play (RTP) is possible after microfracture, but there is little data on subsequent career longevity [1]. Furthermore, some studies report that prior microfracture reduces the success of other treatments such as ACI [5, 16, 17, 21, 22]. In view of this, there is less enthusiasm now for microfracture than there was previously [5].

The purpose of this retrospective study of a consecutive case series was not only to determine RTP rate and factors affecting RTP, but also to document career level and longevity in elite sport after microfracture.

We hypothesised that microfracture for full‐thickness chondral lesions in the knee leads to high RTP rates.

## MATERIALS AND METHODS

A retrospective review of a consecutive series of microfracture procedures undertaken in elite athletes by the lead author between 2011 and 2020 was carried out. Approval to complete the study was given by the institute involved in line with UK Health Research Authority guidance.

Patients were included if they were aged over 16 years, played elite sport, and received microfracture to one or more full‐thickness chondral lesions of the knee [26]. All athletes who had concomitant ligament reconstructions, patellar stabilisation procedures, or had previous surgery for a chondral lesion were excluded. Cases in which prior meniscectomy had been undertaken were included if the chondral lesion appeared acute on magnetic resonance imaging (MRI) and at arthroscopy, for example, sharp edges, blood in the lesion, fresh‐looking loose bodies. Cases of chondral damage that seemed chronic and secondary to attrition from meniscal deficiency were excluded. Elite athletes were defined as those who are paid to perform their sport or those who participate at the national/international level in an amateur sport.

Prior to microfracture surgery, all patients underwent a full clinical knee examination and MRI scan. Data regarding demographics, injury, sport played, clinical and operative details, complications and any subsequent surgery were collected from the clinical records. RTP and continued sport participation details were found on publicly available databases such as www.fbref.com or transfermarkt.co.uk for football, and also direct communication with the players' medical teams. Factors including age, body mass index (BMI), sport, injury mechanism, concurrent meniscal surgery and lesion size, technique of microfracture, and number of lesions were analysed to determine their effect on RTP and career longevity. It was not possible to find the cause of retirement from the sport concerned, and therefore could relate to problems apart from the knee chondral damage.

In lesions less than or equal to 2 cm^2^ the indications for surgery were: (1) failure of resolution of symptoms after non‐operative treatment (e.g., aspiration of effusion and injection of hyaluronic acid plus rehabilitation) for a minimum of 12 weeks; (2) lesions that caused loose bodies requiring arthroscopic removal and the lesion was simply stabilised, but symptoms remained at 8 weeks later.

### Surgical technique

The technique of the senior surgeon (A.W.) did not change throughout the study duration. At surgery, the lesion was debrided to create stable vertical edges. The base of the lesion was then curetted to remove any remaining patches of articular cartilage, being careful not to damage the subchondral bone. Marrow stimulation was performed using a chondral pick or the MicroFx drill (Stryker™). At the end of surgical intervention, the lesion size was carefully measured and recorded, and for femoral lesions, the arc of contact of the lesion with the tibia was recorded to help fine‐tune rehabilitation for appropriate ranges of motion under load.

### Rehabilitation

The rehabilitation protocol was dependent on the location and arc of contact of the lesion with its opposing articular surface. The principle was to offload the lesion for 6 weeks, during which time a continuous passive motion (CPM) machine was used for 4–6 h each day to stimulate healing. If the lesion was in the patellofemoral joint or the unusual situation on the posterior femoral condyle and not making contact with the tibia in late extension, patients were allowed to fully weight bear with a brace locked in extension. For lesions of the femoral condyle, which made contact with the tibia in the weight‐bearing arc of knee flexion, patients were strictly non‐weight‐bearing for 6 weeks post‐surgery. The CPM range was determined by the arc of motion the lesion was in contact with its opposing joint surface. Static cycling with no resistance was allowed from Week 2, with increasing resistance added from 6 weeks post‐surgery. A formal loaded strength and balance programme started at the beginning of the seventh week and increased in intensity as tolerated. Straight leg raises and isometric quadriceps contractions started immediately post‐operatively. A muscle stimulator was used if available. Closed kinetic chain quadriceps exercises were used from the start of the seventh week, but open chain quadriceps work commenced at the start of the eleventh week after surgery.

RTP was defined as playing at least one match or competing in at least one event at a professional level or at national/international level in an amateur sport [14]. Time to RTP was the time between microfracture and the first professional match. Level of play was determined by the league level, and 2‐ and 5‐year playing rates, and overall career longevity were determined by the match appearance dates found at the time of the study.

### Statistical analysis

Statistical analysis was carried out using Python (v3.11). Categorical variables are expressed as numbers (percentages) and continuous variables as mean (±SD). Differences were examined using Chi‐squared tests for categorical variables and for numerical variables, *t*‐tests were used if they were normally distributed, and Mann–Whitney *U*‐test if they were not normally distributed. Statistical significance was set at *p* = 0.05.

## RESULTS

Sixty‐seven consecutive elite athletes underwent microfracture to the knee between March 2011 and November 2020. All cases could be followed up on. Seventeen patients were excluded: 3 due to previous surgery for chondral lesions and 14 due to concomitant surgery in the ipsilateral knee. Therefore, 50 athletes, 48 male and 2 female, were included in the study. Mean age was 24.7 years (±4.0 years, range: 17–34 years) (Table 1).

**Table 1:** Patient characteristics.

Characteristic	Number of patients, *n* (%)
Age band (years)	
<25	24 (48%)
25 and older	26 (52%)
Sport	
Soccer	30 (60%)
Rugby	13 (26%)
Other (cricket, hockey, hurling)	7 (14%)
Lesion mechanism	
Idiopathic	14 (28%)
Post‐ACL	8 (16%)
Post‐meniscectomy	16 (32%)
Trauma	12 (24%)
Microfracture lesion size (cm^2^)	
≤2	29 (58%)
>2	21 (42%)
Lesion location	
Medial femoral condyle	6 (12%)
Medial tibial plateau	0 (0%)
Lateral femoral condyle	28 (56%)
Lateral tibial plateau	1 (2%)
Trochlea	11 (22%)
Patella	0 (0%)
Multiple sites	4 (8%)
Number of lesions	
1	36 (72%)
2	12 (24%)
3	1 (2%)
4	1 (2%)
Concomitant meniscal surgery	
None	25 (64.1%)
Medial meniscal surgery	2 (5.1%)
Lateral meniscal surgery	12 (30.8%)

Abbreviation: ACL, anterior cruciate ligament.

All patients underwent non‐operative treatment prior to surgery for a minimum of 12 weeks, without significant improvement in symptoms.

Forty‐seven (94.0%) athletes RTP at a mean time of 9.3 months (±4.1 months). The size of the lesion had a statistically significant effect on RTP (*p* = 0.048), but other factors such as age, BMI, sport, mechanism of lesion and concomitant meniscal surgery did not affect it. Out of 50 elite athletes, 37 (74%) returned to their previous level, and 10 (20%) returned to one level lower. Only 6% failed to RTP.

**Figure 1:**
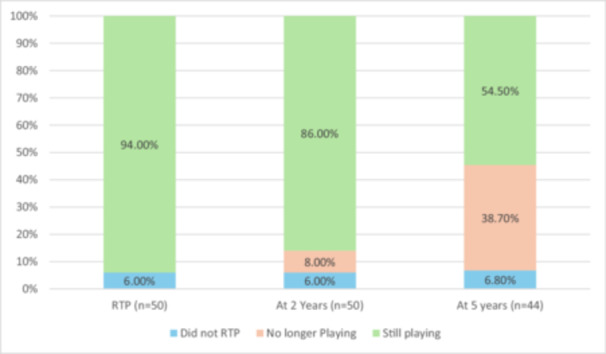
RTP and playing rates at 2 and 5 years.

At 2 years, 43 (86%) of the athletes were still playing. Forty‐four athletes had more than 5 years of follow‐up, and 24 (54.5%) were still playing (Figure 1).

Although the size of the lesion and the number of lesions affected career longevity, no other factors evaluated had a significant effect. At 2 years, only an increasing number of lesions significantly reduced the number of athletes still playing (*p* < 0.001). By 5 years post‐surgery, both size and number of lesions had a significant effect on the ability to continue to play at 5 years (*p* = 0.051 and *p* = 0.002, respectively).

When categorised into lesions of ≤2 and >2 cm^2^, no statistical difference was found regarding RTP time (8.6 months for ≤2 cm^2^ and 9.3 months for >2 cm^2^, *p* = 0.135) and playing rates at 2 years (89.7% for ≤2 cm^2^ vs. 81% for >2 cm^2^, *p* = 0.973) or at 5 years (58.6% for ≤2 cm^2^ vs. 33.3% for >2 cm^2^, *p* = 0.249).

## DISCUSSION

The most important findings of the present study were that following microfracture for full‐thickness chondral lesions in the knees of elite athletes, RTP rates are high (94% with 74% returning to the same level of sports). However, the RTP time is long (mean: 9.3 months). In addition, with time, less athletes remain playing (54.5% at 5 years). Outcome is adversely affected by the increased size and number of chondral lesions.

Only a limited number of studies have investigated factors affecting RTP and career longevity following microfracture in elite athletes [2, 3, 8, 23, 30]. In 2018, in a series of 131 elite athletes (Major League Baseball [MLB], National Basketball Association [NBA], National Football League [NFL] and National Hockey League [NHL]) following primary microfracture of the knee, Schallmo et al. presented an overall RTP rate of 78.6% (MLB 100%, NBA 82.4%, NFL 71.1% and NHL 100%) with a mean RTP time of 9.8 months [29]. The variations between sports indicate that the sports played in any study regarding the treatment of chondral lesions will affect outcomes. Schallmo et al. present the largest series of professional athletes treated with microfracture published. They stated that 67% of the athletes were still playing at 1 year and 43% at 3 years, while overall median survival was 2.8 years [29]. Compared to the present study, they showed lower RTP rates (78.6% overall, 71.1% NFL, vs. 94% in our study) but a similar RTP time (9.8 vs. 9.3 months). The biggest difference is the significantly lower career longevity rates in their series compared to the present study (67% at 1 year and 43% at 3 years vs. 86% at 2 years and 54.5% at 5 years), but the athletes in the two studies played different sports to those in the present study so comparison is difficult. Schallmo et al. did not present data regarding the influence of the lesion size [29].

Richard Steadman popularised the microfracture technique and reported a mean time from surgical intervention to RTP of 13.4 months and an RTP rate of 95% to elite level in professional alpine ski racers [31]. In 2003, he published on 25 professional NFL players with an RTP rate of 76% [32]. However, the factors affecting the RTP were not investigated. Again, the differing results emphasise the importance of results being considered sports‐specific and not comparable to other sports.

In 2009, Riyami et al. [25] presented prospective data including 24 professional football and rugby players. The patient cohort, which was similar to the present study, reported a quicker mean time to RTP of 6.2 months but showed a lower RTP rate of 83.3%. The mean lesion size was 2.0 cm^2^. Only RTP data was presented. In a case‐control study from 2013, Harris et al. matched 41 NBA players who underwent microfracture with an 83% RTP rate to professional basketball at a mean time to RTP of 9.2 months. Furthermore, there was no significant difference in career length between their two cohorts (microfracture vs. non‐injured athletes) [11].

A scoping review of recreational to professional athletes involved in pivoting sports published by Toyooka et al. presented RTP rates from 67% to 93%, compared to 94% in the present study, and time to RTS ranging from 11.8 weeks to 6.5 months after microfracture [33], which reflects the wide variability of practice and experience.

In the present study, the mean time to RTP was 9.3 months, which is long when there is a common perception that microfracture allows earlier RTP compared to ‘regenerative techniques’ such as ACI, which are similar. The RTP time may be adversely affected by the stage of the season the player gets back into full training. Players who are ready to return to full training towards the end of the season may reasonably delay playing in a match until the following season. This results in approximately 3 months being added to the RTP time due to the fixture schedule. In addition, players ready to play are not always selected by their team officials.

Unsurprisingly, and in common with longevity studies after other knee surgeries in athletes, the rates of players still competing decrease significantly over time. In the present study, with a mean age of 24.7 years at surgery, the rate still playing at 5 years is only 54.5%. For a study of the general population, return to activity at a rate as low as this would be viewed as being very poor. However, it is clearly not as easy to return elite athletes to their sport. Furthermore, it is difficult to know how much of this decrease in still playing rate is due to the natural attrition of elite sport participation and how much is related to the injury, especially as the cause of retirement from professional sport was not available in the present study, and athletes' career lengths are often short. In a study of 4117 footballers playing in the top 4 English leagues, assessing overall career lengths and levels of performance to establish what is ‘usual’ in this group of athletes [12]. It was found that careers were longer in the higher‐level players. Using data from this study, for a 25‐year‐old (the mean age in the present study is 24.7 years) footballer, the years of further career expected for a player in the English Premier League, and playing internationally, is 9 years, but for an English Football League 2 footballer, it is only 4 years.

Lesion size is an important issue when it comes to RTP. Lesions >2 cm^2^ are associated with a longer RTP time [11, 19, 34]. The present study could not show differences in RTP times when comparing athletes with lesions ≤2 and >2 cm^2^, but showed that bigger lesions led to reduced RTP rates (*p* = 0.048) and reduced continuing to play at 5 years (*p* = 0.051).

Regarding patients' age, Mithoefer et al. described higher RTP rates in recreational to competitive athletes <40 years [18], while Gudas et al. showed a correlation between patients' age and long‐term outcomes, with the younger patients doing better [10]. However, the cohorts studied were not limited to elite athletes. In the cohort in the present study, age had no significant influence on RTP rate, RTP time, or career length. This may be because the patient cohort of the present study is young (24.7 ± 4.0 years).

It is important to state that there is some evidence that prior microfracture reduces the success of other chondral resurfacing treatments such as ACI in the non‐athlete population [5, 10, 16, 17, 20, 22].

In addition to the retrospective design of this study, further limitations of the present study should be noted. First, no comparisons have been made with other treatment techniques. Toyooka et al. described that rates of RTP in athletes (of all levels) treated with microfracture were not superior in comparison to other techniques. OAT procedures showed the highest RTP rates [33], but it is unknown if these outcomes are comparable to the professional athlete population in the present study (44%–83% RTP in microfracture patients and 87%–100% RTP in OAT patients vs. 94% RTP in the present study) [33].

Another issue is that multiple sports are included. As described above results of outcomes of treating chondral lesions do vary according to the sport played.

In clinical outcome studies, it is customary to report patient‐reported outcome measures' scores (PROMs). However, for both the elite athlete and their team, RTP, level of play and longevity after any injury are far more important measures of outcome than PROMs. While, as demonstrated in the current study, these major aspects of outcome are accurately collected in elite athletes, with close to 100% follow‐up, PROMs are impossible to collect due to lack of compliance in this patient group.

Another limiting factor of the present study is that it only reports on elite athletes, and outcomes are not necessarily applicable to the general public. The quality of, and commitment to, rehabilitation is likely to be higher than in other patient groups, as is the necessity to RTP, and the ability to tolerate chondral damage. On the other hand, the higher demands on athletes' knees test a procedure more and so allow better determination of its efficacy. Also, the follow‐up rate of these patients is 100%.

Furthermore, in the present study, there was no assessment of the ‘quality of healing’ following microfracture by follow‐up MRI or arthroscopy.

Despite these limitations, the study clearly shows that microfracture of chondral lesions in the knee allows elite athletes to return to sport and to continue playing at a professional level, although with diminishing longevity. It also provides information for the player and their medical teams that may help inform decision‐making. These findings are not necessarily in keeping with the popular practice for treatment of symptomatic chondral lesions needing surgery, which has become negative regarding microfracture. The authors of the present study would emphasise that the present study is not aiming to promote microfracture as a treatment for all chondral lesions in all populations, but simply to report the results in the very select patient group of elite athletes and make the point that microfracture is ‘not all bad’ as seems to be a commonly held view. In this unusual patient group, treatment simply aims to restore RTP, hopefully so at the pre‐injury level, and for as long as possible. A much longer‐term outcome is less relevant to this patient group compared to the general population.

## CONCLUSION

The rate of RTP of professional athletes after microfracture in the knee is high. The ability to continue playing reduces over time and is affected by the size and number of lesions requiring microfracture.

## AUTHOR CONTRIBUTIONS

All authors contributed to the conception of the study. *Study design*: David J. Haslhofer, Mary Jones and Andy Williams. *Ethical approval*: Mary Jones, Simon V. Ball and Andy Williams. *Data collection*: David J. Haslhofer, Jobe Shatrov, Mary Jones and Andy Williams. *Data analysis, statistical analysis*: David J. Haslhofer, Jobe Shatrov, Mary Jones and Andy Williams. *Drafting manuscript, tables and figures*: David J. Haslhofer, Jobe Shatrov, Mary Jones and Andy Williams. *Revision manuscript, tables and figures*: David J. Haslhofer, Mary Jones, Simon V. Ball and Andy Williams. The final version was approved by all authors.

## CONFLICT OF INTEREST STATEMENT

Andy Williams has shares/stock in Innovate Orthopaedics, DocComs, and is an Editorial Board Member in AJSM; received research funding and lecture fees, and is a part‐funding salary clinical fellow at Smith and Nephew. Simon V. Ball received research funding and a part‐funding salary clinical fellow at Smith and Nephew. The remaining authors declare no conflicts of interest.

## ETHICS STATEMENT

The authors have nothing to report.

## Data Availability

Data are available from the corresponding author upon reasonable request.
